# Feasibility of virtual reality based training for optimising COVID-19 case handling in Uganda

**DOI:** 10.1186/s12909-022-03294-x

**Published:** 2022-04-13

**Authors:** Paul Buyego, Elizabeth Katwesigye, Grace Kebirungi, Mike Nsubuga, Shirley Nakyejwe, Phillip Cruz, Meghan C. McCarthy, Darrell Hurt, Andrew Kambugu, Joseph Walter Arinaitwe, Umaru Ssekabira, Daudi Jjingo

**Affiliations:** 1 Infectious Diseases Institute, Makerere University, Kampala, Uganda; 2 The African Center of Excellence in Bioinformatics and Data Intensive Sciences, Makerere University, Kampala, Uganda; 3 Office of Cyber Infrastructure and Computational Biology, National Institute of Allergy and Infectious Diseases, National Institutes of Health, Bethesda, MD 20892 USA; 4 Department of Computer Science, College of Computing and Information Sciences, Makerere University, Kampala, Uganda

**Keywords:** Virtual reality, COVID-19, Personal protective equipment, Medical education, Pandemics

## Abstract

**Background:**

Epidemics and pandemics are causing high morbidity and mortality on a still-evolving scale exemplified by the COVID-19 pandemic. Infection prevention and control (IPC) training for frontline health workers is thus essential. However, classroom or hospital ward-based training portends an infection risk due to the in-person interaction of participants. We explored the use of Virtual Reality (VR) simulations for frontline health worker training since it trains participants without exposing them to infections that would arise from in-person training. It does away with the requirement for expensive personal protective equipment (PPE) that has been in acute shortage and improves learning, retention, and recall. This represents the first attempt in deploying VR-based pedagogy in a Ugandan medical education context.

**Methods:**

We used animated VR-based simulations of bedside and ward-based training scenarios for frontline health workers. The training covered the donning and doffing of PPE, case management of COVID-19 infected individuals, and hand hygiene. It used VR headsets to actualize an immersive experience, via a hybrid of fully-interactive VR and 360° videos. The level of knowledge acquisition between individuals trained using this method was compared to similar cohorts previously trained in a classroom setting. That evaluation was supplemented by a qualitative assessment based on feedback from participants about their experience.

**Results:**

The effort resulted in a COVID-19 IPC curriculum adapted into VR, corresponding VR content, and a pioneer cohort of VR trained frontline health workers. The formalized comparison with classroom-trained cohorts showed relatively better outcomes by way of skills acquired, speed of learning, and rates of information retention (*P-value = 4.0e-09).* In the qualitative assessment, 90% of the participants rated the method as very good, 58.1% strongly agreed that the activities met the course objectives, and 97.7% strongly indicated willingness to refer the course to colleagues.

**Conclusion:**

VR-based COVID-19 IPC training is feasible, effective and achieves enhanced learning while protecting participants from infections within a pandemic setting in Uganda. It is a delivery medium transferable to the contexts of other highly infectious diseases.

**Supplementary Information:**

The online version contains supplementary material available at 10.1186/s12909-022-03294-x.

## Background

In the first two decades of the twenty-first century, humanity has witnessed disease outbreaks that have highlighted the danger of chronic vulnerability to infectious diseases, known and unknown. These epidemics and pandemics have resulted in high morbidity and mortality on a still-evolving scale [1]. Among these are SARS CoV, MERS, Ebola, Marburg, cholera, influenza, and current SARS CoV-2 [1]. Uganda is particularly prone to infectious disease outbreaks [2, 3]. Between 2000 and 2016, Uganda reported eight outbreaks caused by filoviruses (Ebola Virus Disease (EVD) and Marburg), more than any other country in the world [4].

Healthcare workers (HCWs), support staff, patients, and visitors to health facilities are at risk of acquiring such infections in healthcare settings as well as from the community [3]. With the current COVID-19 pandemic, 2299 health workers in Uganda have contracted the disease so far, and 28 have died, reported as of 14th June 2021 [5]. To minimize the risk of transmitting these infectious agents, use of infection prevention and control (IPC) practices should be paramount [3]. IPC is a practical, evidence-based approach that focuses on preventing patients, visitors, and HCWs from being harmed by avoidable and preventable infections in a healthcare setting [3]. In Uganda’s health facilities, IPC is still limited as evidenced by widespread noncompliance to hand hygiene measures, poor waste management, lack of isolation protocols, and lack of functional IPC committees [2, 6–9].

Endeavors to improve IPC practices have included several classrooms trainings for HCWs. However, a functional simulation exercise conducted in April 2019 to assess readiness found weak infection prevention and control practices in districts where trainings had been conducted [10]..

IPC training involves not only knowledge but also extensive practical skills training which entails students refining and practicing IPC procedures repetitively. The classroom-training model used may not offer the opportunity for procedural repetitiveness and often does not provide the actual rendition of a highly infectious setting. Furthermore, IPC practical training requires the use of many cleaning consumables and single-use protective gear, which is very wasteful and makes the training sessions non cost-effective. Recognizing that virtual reality (VR) is considered an effective methodology for procedural training, [11] we therefore proposed and piloted the use of VR simulations for IPC training of frontline HCWs in Uganda.

VR is the use of computer technology to create a simulated environment that immerses the user into an experience. It uses dynamic 3D-visualization to actualize a near real-world rendition of the circumstances and context [12, 13]. The technology can be used to create realistic environments or even simulations that would be difficult or expensive to actualize in conventional physical reality. The multi-sensory, immersive experience of VR during the learning process has been shown to improve engagement and mental alertness of both students and teachers [14–16]. Consequently, it speeds up the rates at which individuals assimilate information and increases the extent of information retention [17, 18]. That information assimilation is driven by the technology’s ability to more precisely simulate features and processes, give learners real-time interactive feedback, and give extreme close-up and dynamic multi-perspective views of objects. Accordingly, VR is broadly applicable and has been applied to many different areas of education including technology training [19], natural sciences, history and architecture [19]. It has been described as the learning aid of the twenty-first century [20].

Perhaps medicine represents one of the fields where VR has proven most effective owing to that field’s dependence on elaborate illustrations of anatomic and physiological features [21, 22]. The pedagogical design for medical education involves not only theoretical knowledge but also extensive practical skill training [11]. Practical training, as part of clinical practice, involves a significant amount of standardized procedures that students need to practice and refine repetitively [11]. VR simulation allows learners to experience unlimited use and correction of mistakes through procedural repetition. It also helps mitigate the training deficit that arises out of the prevalent shortage of practical trainers [11]. VR thus facilitates the repetitive drills needed for IPC training, and increases the training opportunities medical practitioners get before facing patients [23]. On the other hand, the simulated VR training allows users to practice interactively within a simulated environment which is difficult and expensive to recreate in the real world [11]. Indeed, studies done on the use of VR in medical education have shown it to produce favorable outcomes in terms of knowledge and skills gained in comparison to classroom instruction [24]. Furthermore, additional studies have demonstrated that VR improves post-intervention knowledge and skills outcomes of health professionals when compared with traditional education or other types of digital education [24]. As a result, VR is increasingly being adopted as a supplementary medium for medical training in Europe and North America [20, 25]. For example, the increasing financial feasibility of VR access has allowed for educational institutions to incorporate the technology into their training at 96% of the universities in the UK [26] and it was forecast to reach over 95 million users in the US by 2022 [27]. Low-cost VR solutions are beginning to emerge, exemplified by one developed to increase surgical oncology capacity and capability [28]. In addition, the American Heart Association (AHA) highlights the potential of immersive technologies to improve resuscitation training, through their ability to enrich learning experiences [28, 29]. VR is also gaining use in rehabilitation therapy because of its ability to bridge the gap between laboratory training and the real-life tasks involved in daily living [30, 31].

However, the feasibility of VR in low-resourced, less endowed biomedical/health education systems with a lower technology exposure and technology culture like Uganda has not been tested partly because of a prior of lack of requisite equipment and VR skilled individuals to develop and manage its platforms. The recent emergence of multiple epidemics in several low resourced environments including Uganda has added to the urgency to test its viability in such environments due to its ability to enable effective training of health workers with a minimum risk of infection. In this paper, we pursue an overall research objective of evaluating the feasibility of such an approach for training health care workers in IPC within the context of an active highly infectious COVID-19 pandemic in a resource limited setting. We do so through two specific objectives: conversion of some modules of COVID-19 IPC classroom curriculum into VR mode and piloting them with a cohort of pioneer participants using an improvised hybrid of VR and 360^0^ videos. The cohort is then used to assess both the feasibility, acceptability and effectiveness of VR in such settings.

## Methods

### Process summary

The work was implemented through a VR-based simulation of animated IPC bedside and ward-training scenarios for critical health workers. The training covered the handling of VR equipment, wearing and stripping of Personal Protective Equipment (PPE), case management of individuals infected with COVID-19 and hand hygiene, all while ensuring maximum safety from infection and improving access to the training.

### VR hardware and software

The VR training sessions were conducted at the ACE Viz Lab facility at IDI. Each participant was fitted with an HTC Vive Pro head-mounted display (HMD) and two hand controllers. The system includes multiple wall-mounted base stations that emit safe, infrared light that is detected by sensors on the HMD and controllers to provide highly accurate room-scale tracking. Each PC has a high-performance GPU to ensure high-resolution optimal VR experience for the user.

Immersive content for the different phases was provided through: (1) Enduvo (Enduvo, Inc. Peoria, IL, US), a VR training platform for the introductory phase with content created by the authors (https://my.enduvo.com/course/6VuYTzTXY.); (2) Humulo (Humulo, Inc., Edgewater, MD, US), a VR training platform for the interactive training for donning and doffing, using a custom module created by Humulo with requirements provided by the authors; (3) SOMA, a platform for creating and consuming VR content in the form of 360^0^ videos, with content created by the authors.

### COVID 19 IPC curriculum adaptation and platform preparation

The COVID-19 IPC Virtual Reality course and curriculum were developed using the updated Ministry of Health COVID-19 IPC classroom/in-person course as the guide [32]. Essential topics and skills were extracted and used to design a customized preliminary curriculum map with particular attention to skills amenable to VR renditions. The curriculum map was then strengthened with additional feasible simulations and renditions, such as that of the COVID-19 virus particle and its anatomy. The resulting curriculum map covered five modules; Introduction to VR equipment, Introduction to the SARS-CoV-2 virus, Infection Prevention and Control, Disinfection and waste management, and COVID-19 case management (Table 1). The updated IPC training materials corresponding to each curriculum module were then transformed into VR space as artifacts through an artifact design, coding and launching step. Here, each artifact representing entities like persons, PPE gowns, sanitizers, beds and other objects within the training environment was separately designed and implemented using VR software. Each artifact was then supplemented with requisite activities by adding to it dynamic motions and gesticulations. Narratives were then synchronously added to the dynamic artifacts via voice-over narrations and adding text captions. The aggregation and alignment of those three information channels: the artifacts, activities, and audio narrations, were enabled by the relevant *Enduvo* VR software platform functionalities and consequently embedded into the VR platform. This content was then organized and arranged into various virtual reality modules or interactive scenes, which represent steps and protocols of IPC and PPE practice.

**Table 1:** VR Curriculum Map

Module and sessions	Training duration
**Week 1: VR Platform**	
**1. Introduction to VR Equipment**	**Total duration 5 min**
**2 . Introduction to Sars-Cov-2** 2.1 Understand what COVID-19 is 2.2 Describe the pathophysiology of COVID-19 2.3 Understand the clinical presentation of COVID-19 2.4 Understand screening and triaging of COVID-19 patients	**Total duration 20 min** 5 min 5 min 5 min 5 min
**3. Infection Prevention and Control** 3.1 Understand what IPC is 3.2 Understand the importance of hand hygiene in IPC 3.3 Understand respiratory hygiene 3.4 Describe Personal Protective Equipment	**Total duration 20 min** 5 min 5 min 5 min 5 min
**4. Disinfection and Waste management** 4.1 Understand the three levels of decontamination in COVID-19 prevention 4.2 Understand the waste management process in a COVID-19 situation	**Total duration 10 min** 5 min 5 min
**5. Understand COVID-19 case management** 5.1 Patient management principles	**Total duration 5 min** 5 min
**Week 2: 360**^**0**^ **videos on** ***SOMA***^a^ **platform**	
**1. Performing hand hygiene** 1.1 Alcohol based hand rub 1.2 Soap and water	**Total duration 10 min** 5 min 5 min
**2. Personal protective equipment** 2.1 Gloving and de-gloving 2.2 Respiratory hygiene (demonstrating mask use) 2.3 Donning coverall 2.4 Donning gowning 2.5 Doffing of coverall 2.6 Doffing of gown	**Total duration 30 min** 5 min 5 min 5 min 5 min 5 min 5 min
**3. Case Management** 3.1 Management of Confirmed COVID-19 case at ETU or level II isolation unit	**Total duration 5 min** 5 min

^a^SOMA is a platform to host 360 degree videos that we developed locally

### Pre-piloting

Using the developed VR content, a pre-pilot training of the Infectious Disease Institute (IDI) and MoH IPC expert staff was conducted to gain prior feedback and assessment of the course. This enabled fine tuning the course before it was rolled out to the field clinicians. It was conducted on 10 participants over a 1-week period. Insights gained from this exercise included recognizing the need to continuously review and update content to align with current best COVID-19 IPC practices since WHO and CDC kept updating recommended preventive measures over the course of the pandemic, integrate safety measures during use of the shared VR equipment and improve quality of the 360^0^ videos. To take care of these shortcomings, we revised and updated the content, procured fluid resistant head nets, eye masks and alcohol hand rub for each VR station. We also re-made some of the 360^0^ videos to improve quality.

### Piloting

**Fig. 1:**
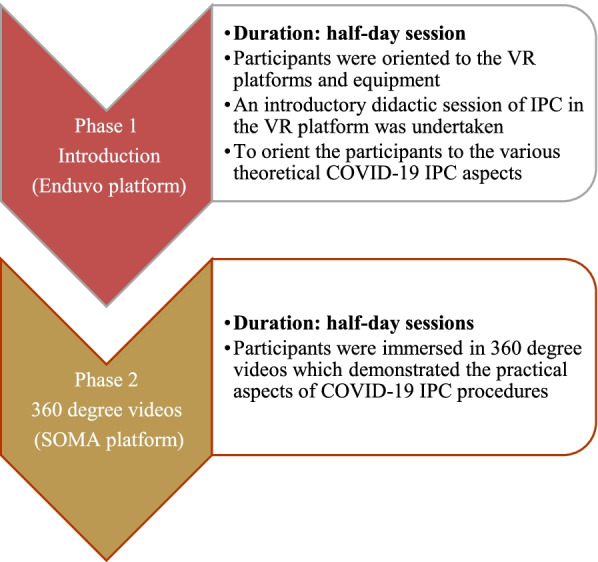
Phase one and two of the pilot training

Having incorporated solutions to shortcomings raised at the pre-pilot level, the exercise was then rolled out to field clinicians as the substantive pilot training. Field clinicians constituted a multidisciplinary team including medical officers, nurses, laboratory, clinical officers, pharmacists and epidemiologists. The pilot training of field clinicians was conducted in two phases for a period of two weeks with a ratio of 1 VR instructor to two participants a day. Phase one consisted of COVID-19 IPC course theory in the immersive Enduvo VR platform for a duration of 1 week and Phase two comprised practical training using 360^0^ videos to complement phase one - for a period of one week. The successful implementation of the second phases (Fig. 1) set the stage for a more fully immersive VR third phase.

#### Phase 1

The introductory phase of this course served to orient participants to the VR platforms and equipment and used a didactic approach to cover the theoretical principles of COVID-19 IPC. Each group of participants received a demonstration of the process of navigation of the Enduvo VR platform showing how to load sessions, navigate between sessions, take a break, do assignments and submit assignments. This phase was delivered by course instructors, who provided both instructional and troubleshooting support to the participants in cases of device or procedural glitches during the course.

Once the participants were comfortable with navigation and manipulation of the platforms, they covered the didactic introductory sessions in the VR platform to orient them to the various theoretical underpinnings of COVID-19 IPC. That content is publicly available in the Enduvo app at https://my.enduvo.com/course/6VuYTzTXY and can be used with or without a VR headset. These were eleven short, objective-guided topics, each with five minutes of run time. However, the actual topic duration was variable as it was self-paced with individual participants having room to pause, forward, rewind and navigate the embedded VR artifacts (Table 2) and videos for their own clarity. At the end of each objective session a multiple choice assessment was undertaken to assess knowledge acquisition. This phase took half a day for each trainee.

**Table 2:**
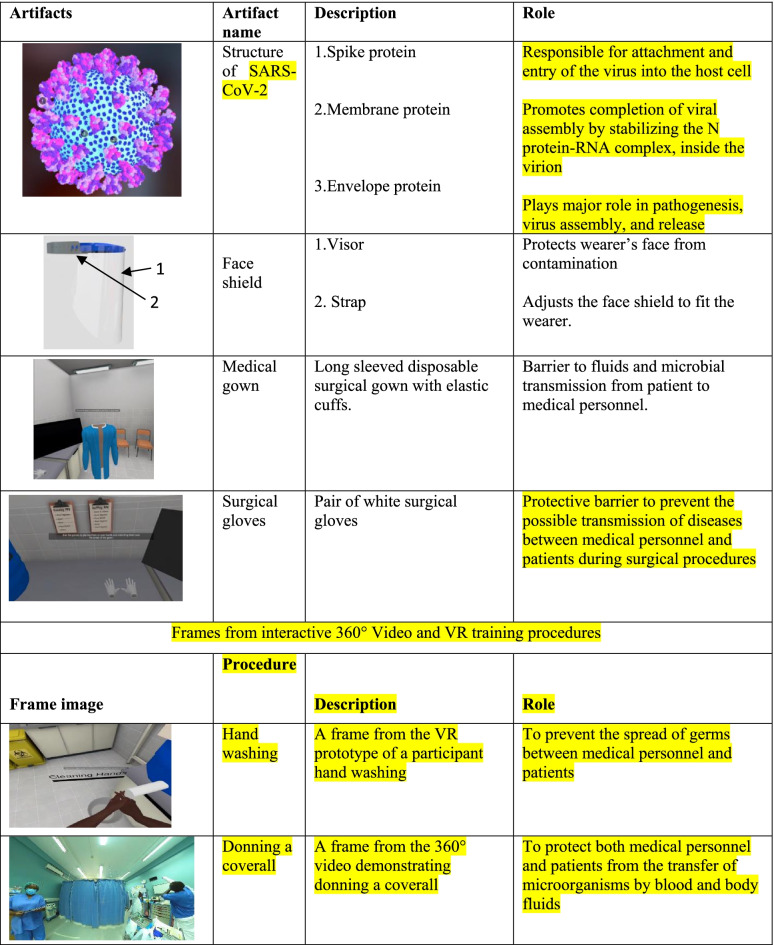
3D VR Artifacts and frames from 360^0^ video and dynamic VR prototype.

#### Phase 2

During this phase, participants covered the practical aspects of COVID-19 IPC and case management. This was done through demonstrations of the procedures using 360^0^ videos in combination with a full-immersion VR prototype for hands on experience (Table 2). The 360^0^ videos helped participants to view pre-recorded IPC and case management scenarios in an enhanced 3D experience which affords them a better and near-real engagement with the scenario (Additional file 1).

This phase involved health workers exploring the 360^0^ VR videos several times in the VR lab until a required level of confidence in performing the task was achieved. At the end of each session an objective assessment in the form of a knowledge based multiple choice question was undertaken to assess knowledge acquisition. Finally, participants were given a demonstration of a full immersion practical VR prototype that guided them through actual practice of IPC activities. This was implemented through a collaboratively developed VR prototype in which each IPC procedure is repeated until the desired level of competence is achieved. It included an immersive, dynamic and interactive VR environment which flags participants every time they get a step wrong through an audio cue and doesn’t let them proceed until they have perfected the procedure (Additional file 2). This phase took half a day for each trainee. The lab is actively procuring equipment to support the full interactive conveyance of this phase.

### Training structure

The phases were delivered as group sessions consisting of a maximum of six participants each to meet social distancing standards. There were 10 such groups of six participants, with two groups attending the course each day. Each cohort attended phase 1 for half a day in the first week and phase 2 for half a day in the second week. All the sessions were self-paced, and each trainee had the opportunity to iteratively go through each session until they had grasped the content. A VR technical assistant was present to guide the participants throughout their sessions.

### Class size

We targeted to train 60 frontline health care workers practicing in the IDI supported health care facilities within Kampala and Wakiso districts. The sample size and cohorting was determined by the six available training stations within the laboratory, all of which are over four meters from each other. As each group of six participants needed half-day for training, the laboratory could only handle 12 participants a day. Accordingly, in 5 days of the work week, the lab could handle a maximum of 60 participants with appropriate social distancing. Coupling the content with 3D web technology and internet connectivity would allow for scaling the training by orders of magnitude, especially in cases where prospective participants have appropriate devices like smart phones to receive 3D web content.

### Course evaluation

This VR training was evaluated using three broad approaches. In the first approach, learning outcomes in terms of knowledge acquisition and knowledge retention by participants was assessed by comparing similar outcomes in previous cohorts that were trained using the classroom instructional model. The second approach was an experiential assessment designed to gauge the experience of the participants, particularly because this is a new pedagogical approach and technology that many were experiencing for the first time. In this assessment, an online individual survey form was used to evaluate the course. The objective of this survey was to obtain participants’ feedback on the overall implementation of the course including strengths, weaknesses, and recommendations for the various components to inform improvements to subsequent courses. In the survey, we used a Likert scale questionnaire with – number of choices ranging from strongly agree to strongly disagree. The survey consisted of a set of eight multiple-choice questions and four open-ended questions that gave participants an opportunity to express their views in detail.

## Results

The exercise resulted in a VR-based IPC curriculum map (Table 1), corresponding VR and 360^0^ content, 6 practiced VR instructors, corresponding VR and 360^0^ content and a pioneer cohort of 52 well trained frontline health workers.

### Trained cohort diversity

**Fig. 2:**
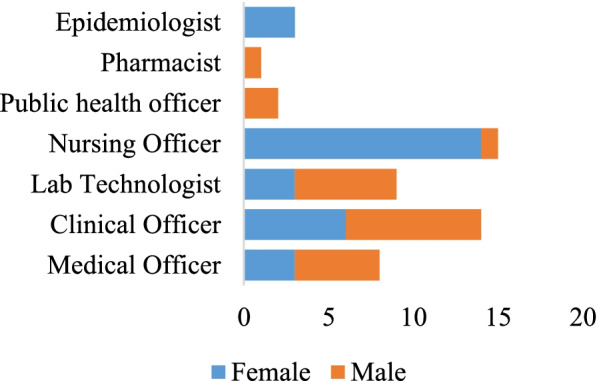
Gender and vocational attributes of participants

Fifty-two frontline health workers participated and completed, with 27 (60%) from government institutions, 23(44%) from a non-government organization (NGO) and 2 (3.6%) from private facilities. The majority of participants were females at 31(56.4%) and males were 24 (43.6%) (Fig. 2). The vocation representation included nurses at 15 (28.9%), followed by clinical officers at 14 (26.9%) laboratory officers 9 (17.3%), Medical Officers 8 (15.4%), Public Health Officers 2 (3.9%), Pharmacists 1 (1.9%), and Epidemiologists 3 (5.8%) (Table 3). The average age was 34.6 with the youngest at 25 and oldest at 60, and a median age of 31. Taken together, the participants constituted a professionally diverse cohort regarding gender, age, type of institution of employment and vocation.

**Table 3:** Vocational distribution of participants among types of institutions

Characteristics of participants	n	%
Occupation (*n* = 52)		
Medical Officer □	8	15.38
Clinical Officer △ □	14	26.92
Nursing Officer △ □	15	28.85
Public Health Officer △ □	2	3.85
Lab technologist △ □ ○	9	17.31
Pharmacist △	1	1.92
Epidemiologist △	3	5.77

**△** ^government organization^ **□** ^Non-government organization^ **○** ^private organization^

### Knowledge and skills acquisition are feasible in VR based training

**Fig. 3:**
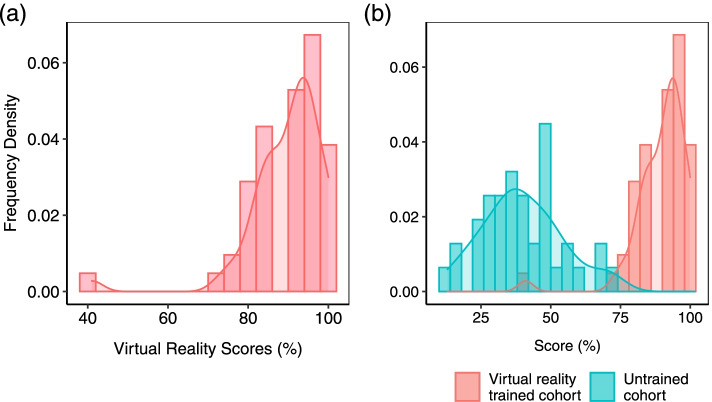
Score comparisons. **a** Distribution of scores for 52 individuals after undergoing VR training. **b** A comparison of the bottom baseline of an untrained cohort (blue bars) with a comparable VR-trained cohort

The completion rate of the course was 98.1% (52 participants). The highest score was 100% in both phases with the lowest being 41% in phase 1 and 33% in phase 2. The average score was 90 and 79.9% in phase 1 and phase 2 respectively. These relatively high scores, where the overwhelming majority of participants scored above 80% (Fig. 3a) suggest the effectiveness and feasibility of VR as a medium of medical training in this low resourced setting. Indeed, when checked against the bottom baseline (a comparable cohort of untrained individuals), there is a clear and significant improvement by way of skills and knowledge gained (Fig. 3b) *p-value = 4.0E-35*.

### Qualitative experiential assessment

**Fig. 4:**
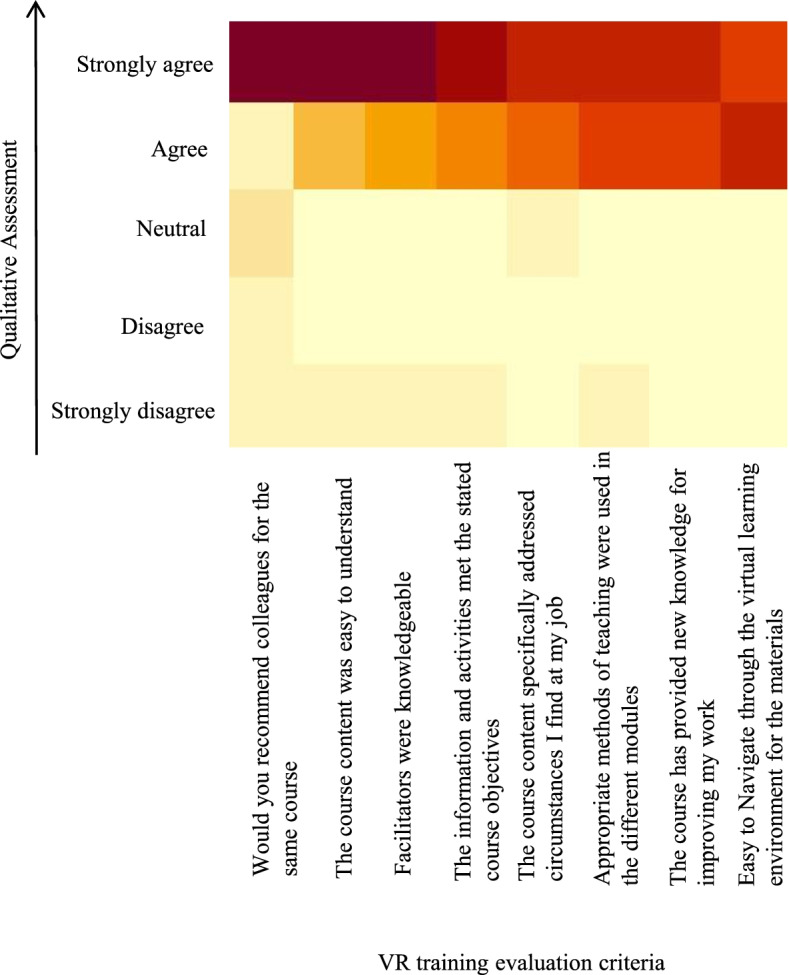
Heatmap depicting extent of approval for the VR training as assessed from survey of participants based on eight criteria (X-axis). Considerably higher numbers of participants (brown) strongly approved of the training relative to those who disapproved (yellow)

Feasibility also needed to be assessed in terms of the levels of comfort with the technology by the participants, which impacts their ability to learn through it as well as its likelihood for acceptance as a training platform by the wider community of health workers. This assessment was done through seeking qualitative feedback from the participants. We received feedback from 80% of the participants, covering whether they felt it met their course expectations, whether it was navigable, whether they felt comfortable learning from it and if they would recommend it to colleagues (Fig. 4). Overall, 58.1% strongly agreed that the information and activities met the course objectives and 65.1% strongly agreed that the course content was easy to understand. Over 90% agreed that the methods for training used were very good and 46.5% of those strongly agreed that appropriate methods were used for the pilot. 46.5% strongly agreed that it was easy to navigate the VR environment for the materials and 97.7% of the participants strongly indicated willingness to refer the course to a colleague (Fig. 4). As might be expected, the younger participants and those with preexisting expertise in other related technology found it easier to navigate the platform and hence complete the course faster. Similarly, participants with visual challenges expressed extra concerns on visibility of the artifacts. In the open-ended questions, participants echoed that the VR mode of learning was more focused, because there was no need to write, they had control of their learning in a single space, there was better concentration in the virtual environment with no interruptions, and it was more practical while utilizing few resources like PPE and stationery. Taken together, the results of these qualitative assessments reveal a net positive experience with the platform and suggest a likelihood for its acceptance as a channel of learning among frontline health care workers in Uganda.

### VR-based training is a comparatively competitive medium of training

**Fig. 5:**
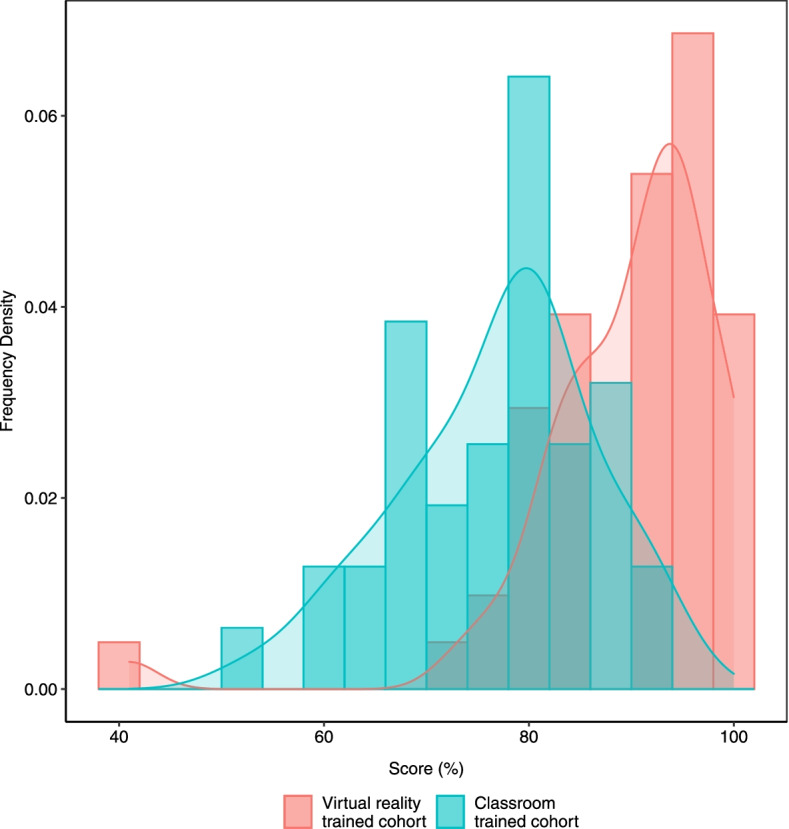
Comparison of the classroom-trained cohort (blue bars) with the VR-trained cohort (red bars)

Having established the viability of VR training for frontline health workers in the context of COVID-19 in a low-resourced environment, we sought to establish how it compares as a pedagogical medium with the pre-existing method of classroom-based instruction. To do that, we drew a similar cohort from health workers we had previously trained using classroom instruction. The cohort was comparable to the VR-trained individuals in terms of the number of participants, their gender distribution, age distribution and vocational background as well as topics covered during the training. As expected, the distribution of scores for both classroom instruction and VR trained cohorts was higher than the bottom baseline of the untrained cohort (Fig. 3b). However, despite a partial overlap, the scores for the VR-trained cohort were statistically significantly higher than those of the classroom-based cohort (Fig. 5) *(p-value = 4.0E-09)*. That difference in learning outcomes suggests VR training has a comparative advantage relative to classroom instruction for COVID-19 IPC procedures among frontline health workers in Uganda. This result is also in line with previous findings showing VR to compare favorably with traditional training methodology [17, 24].

## Discussion

One of the major motivations of this study was to determine the feasibility of VR training in resource-constrained environments. This training piloted virtual reality technology as a new pedagogical approach that presents an alternative training medium for improving competence and optimizing safety of COVID-19 handling and case management in Uganda. The high scores on the post training test for knowledge and skills acquisition attest to the effectiveness and feasibility of the technology for medical training in a low resource setting. This is in agreement with studies in highly resourced settings that have shown that knowledge gain and skills acquisition using VR training approaches was non-inferior to traditional classroom training models [26].

Constraints to VR training in this low-resourced environment included limitations in terms of technology, a paucity of skilled instructors and the lack of a trainee cohort with prior exposure to VR technology. With the improvisations of supplementing VR with 360^0^ videos, we developed a hybrid platform that enabled training even without the full VR complement owing to absence of the latest VR headsets with optimal functionality, and limited VR programming capacity. Furthermore, time was invested in training a critical mass of instructors to learn the use of the hardware and its appropriate and optimal deployment in training sessions. That training helped mitigate the constraint of a paucity of well qualified instructors. Equally important, to accommodate the fact that participants had no prior exposure to VR, the curriculum was designed to begin with a module that brings the participants up-to speed with the use of VR equipment. Finally, the modules were paced and timed to fit within the available training time while also maintaining quality of learning.

As the VR technology matures by way of innovations in hardware and programming platforms, and becomes cheaper, the existing shortfalls will continue to diminish. This will increase its potential as a training medium by enabling a more interactive 3rd phase consisting of more learning artifacts, with more activities and types of interaction within the VR environment. Additionally, its packaging in short modules of 5–10 min and its being amenable to self-pacing should improve learning outcomes while reducing training time. Furthermore, the improved learning outcomes in comparison to classroom instruction that we demonstrated in the results section has been seen in other contexts that found it either equivalent [24] or superior [17]. Its residual cost-related challenge of being scaled to train more health workers in low resourced settings could be mitigated with the use of cheaper cardboard headsets [33] and conversion of the content to web-based 3D experiences as a more broadly accessible alternative to reach a wider pool of participants.

## Conclusion

The results of this VR training pilot indicate that VR can be an effective medium for medical education in Uganda, and merits further investigation with a larger cohort*.* The authors believe that VR has the potential to become a common component of training, diagnosis, illustration, and enhanced communication.

## Supplementary Information

**Additional file 1.** Donning of a coverall. A pre-recorded 360^0^ video of donning a coverall, a procedure in Infection Prevention and Control (IPC).

**Additional file 2.** Donning and doffing Personal Protective Equipment (PPE) in immersive VR. A VR prototype of an immersive, dynamic and interactive environment in which participants practice IPC procedures including handwashing, donning and doffing of PPE. Participants are flagged every time they get a step wrong through an audio cue and the prototype doesn’t let them proceed until they have perfected the procedure.

## Data Availability

Data generated or analysed during this study are included in this published article and its supplementary information files. Auxiliary data is available from the corresponding author on reasonable request.

## Abbreviations

CBDS: Community Based Disease surveillance
CCHF: Crimean Congo Hemorrhagic Fever
EVD: Ebola Virus Disease
GPU: Graphics Processing Unit
HCWs: Health Care Workers
HH: Hand Hygiene
IPC: Infection prevention and Control
MoH: Ministry of Health
NIH: National Institute for Health
OCICB: Office of Cyber Infrastructure and Computational Biology
PPE: Personal Protective Equipment
RIF: Research Innovations Fund
UK: United Kingdom
UNHLS: Uganda National Health Laboratories Services
USA: United States of America
UVRI: Uganda Virus Research Institute
VR: Virtual Reality
